# Auscultation

**Published:** 1881-10

**Authors:** 


### AUSCULTATION

[From Doctor Hall's Family Doctor Book]

IS listening to the sounds given to the ear when it is laid on the chest, whether over the lungs or the heart, to ascertain whether they are in a healthy or diseased condition. To do this with advantage, the person must first know what are the natural, healthful sounds of these organs.

Something can be told by an experienced and quick medical ear, whether certain diseased conditions exist in the bowels, by placing the ear over the belly. But as actual heart disease is comparatively rare, auscultation is mainly applied to the chest, to ascertain the condition of the lungs, whether they are consumptive or not. Consumption seldom exists on both sides of the lungs at the same time, hence if the sounds given out on corresponding spots of the chest are the same, the presumption is that there is no consumptive decay.

Consumption nearly always commences at the top of the chest, immediately under the collar bone. If the sound given to the ear is precisely the same at corresponding parts, the presumption is there is no consumptive decay in either.

If in either of the above cases the pulse is uniformly about seventy beats in the minute, consumption is absolutely impossible.

When the air is drawn into the lungs in their healthy condition, a distinct sound is given, something like the gentle moving of the air in the tops of the trees on a summer's day. This is not so distinct in health when it comes out.

If the sound is more distinct coming out than in going in, there is something wrong. If that sound is blowing, or like blowing into a wide-mouthed vial, then there is a " cavity " in the lungs, which means that there is consumptive decay, which, if it could be seen, would be like a hole made in cheese by mice eating into it; it would be an empty cavity.

But suppose in drawing in a breath, or in going out, a sound is heard as when blowing through a quill or other tube into a thick fluid, as syrup or molasses, that is proof that the lungs have decayed away at that spot, and that the cavity is partially filled with decaying lung-substance in the shape of thick, yellow matter, just like that which is spit out mornings, or during the night, meaning that the person is in the last and hopeless stages of consumption.

There are cases in which the family or friends of the invalid must be thrown upon their own judgment, when it is indispensable that they should decide for themselves, as when a physician cannot be had, or implicit confidence cannot be imposed in him. It may be that the question is to be decided whether business should be given up, or serious sacrifices made to raise means to take a long journey in search of health; or it may be a question of dying from home among strangers, or in the bosom of one's family.

There are many cases of consumption where persons of ordinary intelligence can decide correctly themselves, thus; take a healthy man into a quiet room, throw back the collar of the coat, lay your ear flat on the vest, hold your own breath so as to make no noise, and listen to the sounds given out in a few inspirations and expirations; then lay your ear on the breast, in the same way, and in the same spots of the other as in the healthy man; if the sounds given out are pretty much the same in both persons at each point in the chest in front, or at the points of the shoulder blades in the rear or between the shoulder blades, where alone the lungs are fastened to the body, then, whatever may be the matter with the patient, there can be no consumptive decay. But if you hear the blubbering sound there is decay, there is a cavity, and that cavity is partly filled with yellow matter, the result of decayed lung-substance; if with this the pulse is among the nineties or over, it is confirmed consumption in its last stages, and the man must die; except that if there be very great energy of character or a great force of will, one in a thousand can live down the disease, and live on for a number of years, and finally die of some ordinary malady.

Sometimes in laying the ear on the chest there is no more sound than if it were laid against a wall; this means that the lungs at that point are "solidified," that the little air-cells of the lungs, of various sizes, from a pin's head to a pea, are filled up with tubercles, which are the seeds of consumption; or with blood, as in inflammation of the lungs, which is the same as pneumonia; or some other material, meaning in each case a dangerous condition of the lungs, always giving a symptom which is never by any possibility absent—shortness of breath.

In asthma, the sounds given out are of a wheezing, whistling character, as of a multitude of chirruping birds. There are other sounds given out by the lungs in different states of health and disease, but they are of minor importance, and not so appreciable by the inexperienced, nor can their meaning be decided upon except by those who have had large and long experience.

